# Hospitalizations for ambulatory care sensitive conditions as an indicator of access to primary care and excess of bed supply

**DOI:** 10.1186/s12913-019-4098-x

**Published:** 2019-04-27

**Authors:** Agnus M. Kim, Jong Heon Park, Tae Ho Yoon, Yoon Kim

**Affiliations:** 1 Department of Health Policy and Management, Seoul National University College of Medicine, Seoul, Republic of Korea; 2 Big Data Steering Department, National Health Insurance Service, Wonju, Republic of Korea; 3 Department of Preventive & Occupational Medicine, School of Medicine, Pusan National University, Pusan, Republic of Korea; 4 Institute of Health Policy and Management, Medical Research Center, Seoul National University, Seoul, Republic of Korea

**Keywords:** Ambulatory care sensitive condition, ACSC, Hospitalization, Geographic variation, Beds, Primary care, Korea, Socioeconomic factors

## Abstract

**Background:**

Ambulatory care sensitive conditions (ACSC) hospitalization is a widely accepted measure of the access to primary care. However, given its discretionary characteristics, the ACSC hospitalization can be a measure reflecting the influence of hospital bed supply. In Korea, where the quality of primary care and oversupply of hospital beds are coexistent concerns, ACSC hospitalization can be used to examine the impact of both factors. This study was performed to investigate the ACSC hospitalization rate as a measure of the hospital bed supply as well as access to primary care.

**Methods:**

Data were obtained from the National Health Insurance Database for 2015. We calculated the age-sex standardized hospitalization rates for ACSC in the total population and crude rates of ACSC hospitalization for three different age groups in 252 districts in Korea. We calculated the variation statistics of ACSC hospitalization rates, and we estimated a linear regression model to investigate the factors for ACSC hospitalization.

**Results:**

There was a very high geographic variation in ACSC hospitalization rates. Higher density of primary care physicians was associated with a decreased ACSC hospitalization rate while a higher density of hospital beds in small to medium sized hospitals was associated with an increased rate. The deprivation index score had a strongly positive association with the ACSC hospitalization rates.

**Conclusion:**

ACSC hospitalization, while being a negative index of primary care access, can also be a measure indicating the impact of the hospital bed supply, and it is still a valid measure of the disparity of health care, the original motivation for this topic.

**Electronic supplementary material:**

The online version of this article (10.1186/s12913-019-4098-x) contains supplementary material, which is available to authorized users.

## Background

Hospitalization for ambulatory care sensitive conditions (ACSC) is a widely accepted measure of the function of primary care. Theoretically, as ACSC hospitalization is preventable with a proper supply of “ambulatory care”, it is considered to be a negative index for primary care. Access to primary care, indicated by the number of general practitioners [1–3] or socio-economic conditions [4–6], has generally been shown to have a negative impact on the ACSC hospitalization, although the impact of each variable was not universal and depended on the way primary care was delivered and the overall level of access to primary care among the populations [7–9].

ACSC hospitalization, on the other hand, can also be an indicator of the influence of hospital bed supply. This is due to the discretionary characteristics which are inherent in the concept of ACSC as well as in hospitalization. ACSC is a group of conditions whose treatments are in the spectrum from outpatient to inpatient care, and choosing the location in the spectrum can be a discretionary decision depending on the physician’s judgement. A high degree of geographic variation in ACSC hospitalization reflects its discretionary characteristics [10]. Meanwhile, the discretionary characteristics of hospitalization itself have been well documented in the positive relationship between hospitalization and hospital bed supply [11–13], and this variability of hospitalization rate due to hospital bed supply suggests that the hospitalization decision can be arbitrary. The room for discretion, in the diagnoses of ACSC and hospitalization, makes the hospitalization due to ACSC more sensitive to hospital bed supply. As ACSC hospitalization has been mainly investigated in terms of primary care, hospital bed supply did not receive much attention, and its impact on the ACSC hospitalization remains less investigated [3, 14].

The current health care system in Korea is in an interesting condition for study of the effects of hospital bed supply as well as access to primary care on the ACSC hospitalization rate. Korea has achieved universal health coverage based on a government-run single-payer and private-oriented health care provider system in a short period of time [15]. Due to a haphazard increase in the number of health care providers during a short period and the long-term maintenance of a state where mixture of roles among health care providers was common, primary care in Korea has not been clearly defined regarding its supplier and role with no institutionally defined role of the general practitioner. Patients can visit specialists or hospital physicians as well as general practitioners for the first contact [15]. Although this heterogeneity of primary care suppliers has been the focus of debate in Korea, it is unclear whether the current blurry boundary of primary care in Korea is really detrimental to the health of the population. Investigating the relationship between the number of physicians, who are more devoted to primary care, and ACSC hospitalization in Korea can shed light on this issue.

In addition, Korea is in a condition where the impact of hospital beds on ACSC hospitalization can be clearly examined. The health care system in Korea is largely based on a fee-for-service structure. Therefore, each instance of hospitalization is directly related to the profit of the hospitals. This condition means that hospitalization becomes especially sensitive to hospital bed supply. The number of hospital beds in Korea increased sharply over the last few decades as a result of the rapid growth in the number of hospitals, and its density remains one of the highest among countries [16]. Considering the discretionary characteristic of ACSC hospitalization and the oversupply of hospital beds in connection with the fee-for-service structure, the ACSC hospitalization rate in Korea can respond more sensitively to the hospital bed supply.

This study was performed to examine the ACSC hospitalization rates as a measure of the hospital bed supply as well as access to primary care. We analyzed the geographic variation of the ACSC hospitalization rates according to the 252 districts, and we investigated its factors by a regression analysis.

## Methods

### Study population and data

The study population is the Korean people of all ages, who can possibly be eligible for ACSC hospitalization. We used the inpatient claims of the 2015 period of the National Health Insurance (NHI) Database. The NHI database contains claim data for the entire Korean population (NHI beneficiaries: 97%, medical aid beneficiaries: 3% of the total population).

### Outcome variable

The rates for ACSC were calculated according to the 252 districts and were age- and sex-standardized to the Korean resident population of 2015 [17]. Cluster rates were also calculated for three groups: aged 0–14, 15–64, and 65 and older. We selected 16 diagnoses (Additional file 1), which are relevant to Korea in terms of incidence and association with hospitalization, on the basis of the ACSC proposed by the Institute of Medicine in 1993 [18]. The International Classification of Diseases, Ninth Revision, Clinical Modification (ICD-9-CM) codes of the diagnoses were recoded based on the Korean Standard Classification of Diseases codes 6.

### Explanatory variables

We used the two categories of variables as the potential determinants of the ACSC hospitalization. First, regarding the socio-demographic characteristics of a region, we used 1) the deprivation index [19]. The deprivation index score is the summation of the standardized and weighted value of the variables, which indicate socio-economic conditions of a region. We used a total of nine items which cover the property, income, education, and marital status of the population. The details of composing the deprivation index are described elsewhere [20, 21]. The data were acquired from the Population Census 2010 of Korea.

Second, we obtained the variables for the health care supply from the National Health and Medical Service Statistics 2015 [22]. Concerning primary care providers, there is no established definition for general practitioner in Korea, and primary care practice, in terms of the characteristics of visits and patients, is not limited to specific specialties. Therefore, a conventionally used definition, which defines primary care physicians by specialties [23], may not reflect the actual primary care provider in Korea. Considering this, the Korean government proposed 52 simple and minor disease groups (SMDGs) as criteria for defining primary care [24]. Actually, the specialties where the proportion of patients whose major diagnoses belonged to the 52 SMDGs were highest included otorhinolaryngology (the second highest) and ophthalmology (the fourth) as well as conventional primary care-related specialties such as pediatrics (the first) and internal medicine (the third) [22]. Therefore, we operationally defined the primary care physicians as the physicians in the clinics in which the proportion of the visits with 52 SMDGs [15] is over the average (38.3%) of total clinics [22]. To state the influence of the number of primary care physicians on the ACSC hospitalization as distinct from that of physicians overall, we included the number of active physicians as an explanatory variable. For hospital beds, we used two separate variables to differentiate the influence of the number of hospital beds on ACSC hospitalization rates by the size of hospitals. Korean law classifies medical institutions by the health care personnel and the number of beds which belong to the institution. According to the law, the number of required specialties in general hospitals increases based on the number of beds at 300 [25], and the number of beds at 300 is a generally accepted criterion in Korea for establishing health care policy by dividing small to medium sized and large sized hospitals [26]. Considering the differences in the patients’ characteristics, practice styles, and, especially, the disproportionately sharp increase in the number of small to medium sized hospitals in Korea, we differentiated the number of beds by the size of the hospitals to which they belonged: the number of beds in hospitals with less than 300 beds for small to medium sized hospitals and the number of beds in hospitals with more than 300 beds for large sized hospitals.

### Statistical analysis

First, we calculated the variation statistics of hospitalization rates for ACSC, including the coefficient of variation (CV), the ratio of the rate of the area of the 90th to the 10th percentile of the distribution (P90/P10), and the systematic component of variation (SCV). The SCV is a metric used to estimate the true part of variation due to variation across areas by removing the random part of variation due to within-region variation [27–29]. SCV is calculated on the basis of the difference between the observed and expected value, which is calculated by applying the age-sex specific rate of the entire region to each area. Therefore, SCV can be considered as a measure to remove the part of variation caused by the different population structures among the areas. It has been suggested that SCVs greater than 5.4 suggest high variation, and those greater than 10 very high variation [29, 30]. As indicated by Mcpherson et al., the SCV was presented as being multiplied by 100 [28, 31].

Second, to investigate the relationship between the ACSC hospitalization rate and other independent variables, we performed an ordinary least square regression. The regression analyses were performed for the standardized ACSC rates for all ages and crude ACSC rates for 3 age groups. The regression formula is the following:

Hospitalization rate for bacterial pneumonia = β_0_ + β_1_ deprivation index score + β_2_ number of primary care physicians per 10,000 + β_3_ number of practicing physicians per 10,000 + β_4_ number of hospital beds in small to medium sized hospitals per 1000+ β_5_ hospital beds in large sized hospitals per 1000 + ε.

We used SAS version 9.4 (SAS Institute Inc., Cary, NC, USA), IBM SPSS statistics 23 (SPSS Inc., Chicago, IL, USA) for data acquisition and statistical analysis.

## Results

**Fig. 1:**
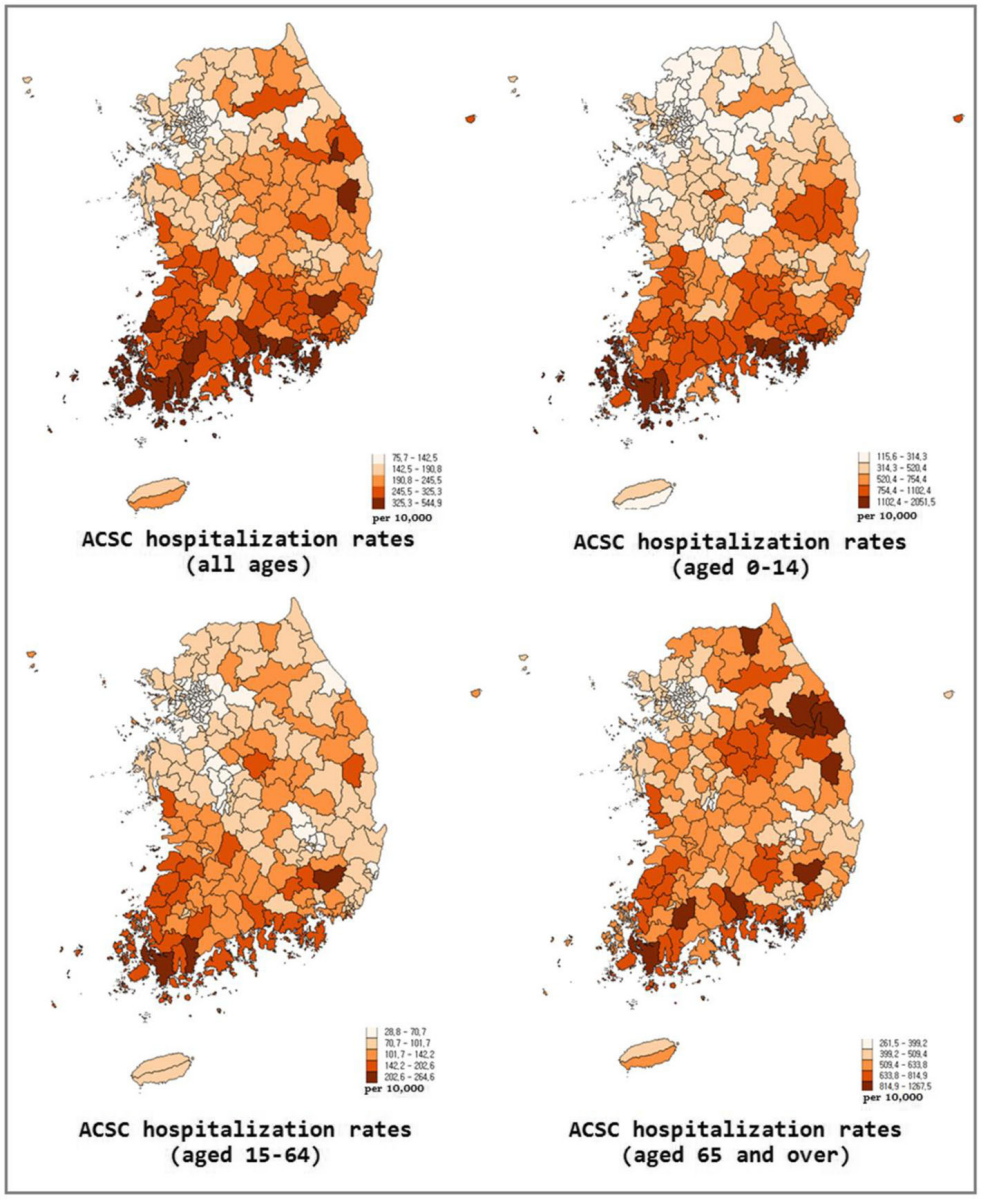
Map of hospitalization rates for ambulatory care sensitive conditions (ACSC) in Korea at the districts level. The left upper map was adapted from the reference [32, 33]

Among a total of 10,002,204 hospital inpatient claims in 2015, excluding those from psychiatric hospitals and clinics for herbal medicine, we identified 921,210 claims (9.2%) for ACSC hospitalization. The national hospitalization rate for the ACSC was 180.8 per 10,000 inhabitants. The most frequent clinical categories among the ACSC were bacterial pneumonia, kidney/urinary infection, angina, and severe infections of ear, nose, and throat (ENT) (Table 1). The average rates for the age groups of 0–14, 15–64, and 65 and over were 562.9, 76.9, and 473.1 per 10,000 inhabitants respectively (Table 1, Fig. 1) [32, 33]. The CV and SCV of the ACSC hospitalization rates for all ages were 0.4 and 16.7 (Table 2) [32, 33]. The variation in the hospitalization rates was the most prominent among the people aged 0 to 14 with CV and SCV at 0.6 and 45.4.

**Table 1:** Absolute frequency and rates of hospitalization for ambulatory care sensitive conditions per 10,000 inhabitants according to diagnostic groups

Diagnosis	All ages	0–14	15–64	65 and over
	No. (%)	No. (%)	No. (%)	No. (%)
Bacterial pneumonia	402,979 (43.7)	232,647 (71.7)	64,523 (22.5)	105,809 (34.1)
Kidney/urinary infection	99,001 (10.7)	16,266 (5.0)	46,318 (16.2)	36,417 (11.7)
Angina	92,993 (10.1)	5 (0.0)	46,475 (16.2)	46,513 (15.0)
Severe ENT infections	70,559 (7.7)	38,038 (11.7)	26,686 (9.3)	5835 (1.9)
Asthma	43,019 (4.7)	11,173 (3.4)	15,671 (5.5)	16,175 (5.2)
Hypertension	42,522 (4.6)	34 (0.0)	16,067 (5.6)	26,421 (8.5)
Cellulitis	39,126 (4.2)	4339 (1.3)	24,657 (8.6)	10,130 (3.3)
Congestive heart failure	32,039 (3.5)	18 (0.0)	5380 (1.9)	26,641 (8.6)
COPD	29,469 (3.2)	662 (0.2)	9948 (3.5)	18,859 (6.1)
Convulsions	19,433 (2.1)	13,563 (4.2)	4546 (1.6)	1324 (0.4)
Diabetes	19,012 (2.1)	450 (0.1)	8658 (3.0)	9904 (3.2)
Epilepsy	18,290 (2.0)	5888 (1.8)	9964 (3.5)	2438 (0.8)
Gastroenteritis	12,668 (1.4)	1535 (0.5)	7577 (2.6)	3556 (1.1)
Total	92,110 (100.0)	324,618 (100.0)	286,470 (100.0)	921,110 (100.0)

*ENT* Ear, nose, and throat, *COPD* Chronic obstructive pulmonary disease. The figures for the group “all ages” (first column) were adapted from references [32, 33]

**Table 2:** Variation statistics of hospitalization rate for ambulatory care sensitive conditions

Diagnosis	Mean	Max	Min	P90/P10	CV	SCV
ACSC hospitalization rate (all ages)	180.8	544.9	75.7	2.5	0.4	16.7
ACSC hospitalization rate (0–14)	562.9	2051.5	115.6	4.4	0.6	45.4
ACSC hospitalization rate (15–64)	76.9	264.6	28.8	2.9	0.4	22.9
ACSC hospitalization rate (65 and over)	473.1	1267.5	261.5	2.1	0.3	9.3

*ACSC* Ambulatory care sensitive conditions, *CV* Coefficient of variation, *SCV* Systematic component of variation, *P90/P10* Ratio of the rate of the area of the 90th to the 10th percentile of the distribution. The figures for the group “all ages” (first row) were adapted from references [32, 33]

Table 3 shows the regression analysis of the ACSC hospitalization rate. The increase in the number of primary care physicians was related to the decrease in ACSC hospitalization rates, and the increase in the number of beds in hospitals with less than 300 beds was associated with the increase in ACSC hospitalization rates. As revealed in the relationship between the deprivation index score and ACSC hospitalization rates, the degree of socio-economic deprivation of an area was associated with the increase in ACSC hospitalization rates. The influence of the independent variables on the ACSC hospitalization rates was similar among the different age groups and was the most marked in the group aged 0 to 14.

**Table 3:** Regression analysis of hospitalization rate for ambulatory care sensitive conditions

	All ages		0–14		15–64		65 and over	
	Coefficient	SE	Coefficient	SE	Coefficient	SE	Coefficient	SE
Baseline (intercept)	201.571****	9.254	479.919****	43.128	95.284****	4.085	566.531****	21.000
Deprivation index	7.075****	1.095	21.883****	5.104	5.413****	.483	13.414****	2.485
Primary care physicians per 10,000 people	−15.498****	3.770	−41.884**	17.570	−8.785****	1.664	−37.509****	8.555
Practicing physicians per 10,000 people	−.179	.502	−1.884	2.339	.234	.222	.402	1.139
Hospital beds in small to medium sized hospitals with no. of beds < 300 per 1000 people)	15.503****	1.397	54.447****	6.511	7.341****	.617	19.295****	3.170
Hospital beds in large sized hospitals with no. of beds ≧300 per 1000 people	.848	2.392	12.862	11.146	−1.269	1.056	−4.558	5.427
Adjusted R^2^	.551		.369		.688		.425	

**p < 0:05 ****p < 0:001

## Discussion

This study was performed to examine the ACSC hospitalization rate as a measure of the access to primary care and the influence of the hospital bed supply by investigating its geographic variation and its factors. Concerning the geographic variations in ACSC hospitalization rates, we assessed the degree of the variation in the ACSC hospitalization rates by comparing it to the variation in the other procedures [31, 34]. We found that the CV and SCV in the ACSC hospitalization rates were markedly higher than the other medical procedures, such as percutaneous transluminal coronary angioplasty, surgery after hip fracture, knee-replacement surgery, cesarean section, hysterectomy, computed tomography scan, and magnetic resonance imaging scan. In particular, given that an SCV greater than 10 can be considered very high [29], our results strongly suggest that there is a very high geographic variation in the ACSC hospitalization rates. This high variation suggests that the ACSC hospitalization rate, the indicator of the access to primary care, is highly subject to the supply factors that are known to be the main reasons for the geographic variation in health care and that there is a considerable geographic variation in both factors.

Our results of regression analysis for the ACSC hospitalization corroborated that supposition. First, we found that the higher density of primary care physicians was associated with the decreased rate of ACSC hospitalization. We could determine the impact of density of primary care physicians on the ACSC hospitalization rate by differentiating it from that of overall active physicians, which could possibly affect the ACSC hospitalization. This result strongly suggests that, despite the unrestricted access to all the levels of health care facilities in Korea, primary care, as revealed by the impact of its access measure on the ACSC hospitalization rate, is still performing an important role for the health of the populations.

Second, the fact that the density of hospital beds was positively associated with the ACSC hospitalization rate supports our initial hypothesis that the ACSC hospitalization rate could serve as a measure for the impact of the hospital bed supply on hospitalization. Especially, our results indicate that the high density of small to medium hospital beds, whose number has been on a sharp rise in recent years in Korea [15], is having a significant influence on hospitalization. The influence of the density of hospital beds on the ACSC hospitalization rate has been investigated by only a few relevant studies, which have shown indefinite results [3, 5, 35]. Our study result clearly shows that, even after adjusting for other important factors for ACSC hospitalization, the density of hospital beds is still an influential factor for ACSC hospitalization.

Third, the strong positive association between the higher deprivation index score and the higher ACSC hospitalization rate supports the results of the previous studies which showed the influence of socio-economic circumstances on ACSC hospitalization. Our results indicate that, despite apparent universal health care coverage, lower barriers to health care access, and high frequency of health care utilization [16, 36], Korea is still suffering from the inequality of health care, especially concerning primary care. Given the concept of the ACSC originating from concern for the indigent [18], the strong relationship between the deprivation index and the ACSC hospitalization shows that this original concern of the ACSC is still valid in Korea 35 years after its inception in the US.

Finally, the influences of explanatory variables on the ACSC hospitalization rates were consistent among the different age groups as well as the total population. However, the impact was the most prominent in the children (0–14) and then in the elderly (65 and above). These results, along with their higher rates for ACSC hospitalization and higher variation, suggest that the vulnerable population, who are more likely to need medical service, are more prone to be influenced by access to primary care and hospital bed supply. This could be due to their relatively higher dependency on the health care provider, which results from their fragile health conditions and lower likelihood of their having and expressing their own opinion concerning medical decisions as compared with young and middle-aged adults.

Our study has several limitations. First, considering that this study is a cross-sectional design, it is difficult to determine the relationships found in our study to be causal ones. Second, as our study is based on a regional unit, our analysis can be subject to the ecological fallacy. Third, the relationship between the hospital beds and the ACSC hospitalization could partly be attributed to the situations specific to Korea involving the sharp increase in and high number of beds and relative ease of being hospitalized. The ACSC hospitalization as a measure of the impact of hospital bed supply should be investigated more in various health care circumstances. In spite of these limitations, our study comprehensively examined the hitherto known variables for the ACSC hospitalization and gave a new significance to the ACSC hospitalization as a measure for the hospital bed supply as well as a monitor for primary care.

## Conclusions

ACSC hospitalization, while being a negative index of primary care access, can also be a measure indicating the impact of the hospital bed supply, and it is still valid as a measure of the inequality of health care. Policy makers should consider using the hospitalization for ambulatory care sensitive conditions for assessing the influence of excess bed supply and socio-economically poor access to care as well as for measuring the performance of primary care. Health authorities in Korea should make efforts to promote primary care while drawing up measures to curb the influence of hospital beds on unnecessary ACSC hospitalization. These efforts should be accompanied by support for the socio-economically disadvantaged populations, who were the original concern for the hospitalization for ambulatory care sensitive conditions.

## Additional file

Additional file 1: Ambulatory care sensitive conditions. The list of diagnoses and the KCD (Korean Standard Classification of Diseases) codes of the ambulatory care sensitive conditions. (DOCX 18 kb)

## Abbreviations

ACSC: Ambulatory care sensitive conditions
COPD: Chronic obstructive pulmonary disease
CV: Coefficient of variation
ICD-9-CM: International Classification of Diseases, Ninth Revision, Clinical Modification
KCD: Korean Standard Classification of Diseases
NHI: National Health Insurance
P90/P10: Ratio of the rate of the area of the 90th to the 10th percentile of the distribution ENT Ear, nose, and throat
SCV: Systematic component of variation
SMDGs: Simple and minor disease groups
