# Supplementary material for: Hospitalizations for ambulatory care sensitive conditions as an indicator of access to primary care and excess of bed supply
Source: BMC Health Serv Res. 2019 Apr 27;19:259. doi: 10.1186/s12913-019-4098-x (PMC6487016; doi:10.1186/s12913-019-4098-x)
Supplement: Supplementary file 1 — Ambulatory care sensitive conditions. The list of diagnoses and the KCD (Korean Standard Classification of Diseases) codes of the ambulatory care sensitive conditions. (DOCX 18 kb) [file 12913_2019_4098_MOESM1_ESM.docx]

**Additional file 1**. **Ambulatory care sensitive conditions**

| Diagnosis | KCD6 | Comments |
| --- | --- | --- |
| Epilepsy | G40 |  |
| Convulsions | R56 |  |
| Severe ENT infections |  |  |
| Suppurative and unspecified otitis media | H66 | Exclude H66 with S5600 (myringotomy) with S5620 (insertion of tube) |
| Acute pharyngitis | J02 |  |
| Acute tonsillitis due to other specified organisms | J03.8 |  |
| Acute upper respiratory infections of multiple and unspecified sites | J06 |  |
| Chronic pharyngitis | J31.2 |  |
| Chronic obstructive pulmonary diseases |  |  |
| Simple and mucopurulent chronic bronchitis | J41 | J20 only with secondary diagnosis of J41, J42, J43, J47, J44.9 |
| Unspecified chronic bronchitis | J42 |  |
| Emphysema | J43 |  |
| Bronchiectasis | J47 |  |
| Chronic obstructive pulmonary disease, unspecified | J44.9 |  |
| Acute bronchitis | J20 |  |
| Bacterial pneumonia |  |  |
| Pneumonia due to Streptococcus pneumoniae | J13 | Exclude case with secondary diagnosis of D57 (sickle cell) and patients under 2 months |
| Pneumonia due to Haemophilus influenzae | J14 |  |
| Pneumonia due to streptococcus, group | J15.3 |  |
| Pneumonia due to Mycoplasma pneumoniae | J15.7 |  |
| Other bacterial pneumonia | J15.8 |  |
| Bacterial pneumonia, unspecified | J15.9 |  |
| Bronchopneumonia, unspecified | J18.0 |  |
| Other pneumonia, organism unspecified | J18.8 |  |
| Pneumonia, unspecified | J18.9 |  |
| Asthma |  |  |
| Asthma | J45 |  |
| Status asthmaticus | J46 |  |
| Congestive heart failure |  |  |
| Heart failure | I50 | Exclude cases with the following surgical procedures: M6551, M6552, HA680, HA681, HA682, Q8080, O0205, O0206, O0208, O0209, O0210 |
| Hypertensive heart disease with(congestive) heart failure | I11.0 |  |
| Acute pulmonary oedema | J81.0 |  |
| Hypertension |  | Same as in congestive heart failure |
| Malignant hypertension | I10.1 |  |
| Unspecified hypertension | I10.9 |  |
| Hypertensive heart disease without(congestive) heart failure | I11.9 |  |
| Angina |  |  |
| Coronary thrombosis not resulting in myocardial infarction | I24.0 | Exclude cases with a surgical procedure |
| Other forms of acute ischaemic heart disease | I24.8 |  |
| Acute ischaemic heart disease, unspecified | I24.9 |  |
| Angina pectoris | I20 |  |
| Cellulitis |  |  |
| Cellulitis | L03 | Exclude cases with a surgical procedure |
| Acute lymphadenitis | L04 |  |
| Other local infections of skin and subcutaneous tissue | L08 |  |
| Diabetes |  |  |
| Other specified diabetes mellitus, with acidosis | E13.1 |  |
| Insulin-dependent diabetes mellitus, with coma | E10.0 |  |
| Other specified diabetes mellitus, with coma | E13.0 |  |
| Unspecified diabetes mellitus, with other specified complications | E14.6 |  |
| Unspecified diabetes mellitus, with unspecified complications | E14.8 |  |
| Unspecified diabetes mellitus, without complications | E14.9 |  |
| Hypoglycaemia, unspecified | E16.2 |  |
| Gastroenteritis | K52.9 |  |
| Kidney/urinary infection |  |  |
| Acute tubulo-interstitial nephritis | N10 |  |
| Chronic tubulo-interstitial nephritis | N11 |  |
| Tubulo-interstitial nephritis, not specified as acute or chronic | N12 |  |
| Renal and perinephric abscess | N15.1 |  |
| Other specified renal tubulo-interstitial diseases | N15.8 |  |
| Renal tubulo-interstitial disease, unspecified | N15.9 |  |
| Urethral disorder, unspecified | N36.9 |  |
| Urinary tract infection, site not specified | N39.0 |  |

KCD. Korean Standard Classification of Diseases
